# Recommendations for implementing stereotactic radiotherapy in peripheral stage IA non-small cell lung cancer: report from the Quality Assurance Working Party of the randomised phase III ROSEL study

**DOI:** 10.1186/1748-717X-4-1

**Published:** 2009-01-12

**Authors:** Coen W Hurkmans, Johan P Cuijpers, Frank J Lagerwaard, Joachim Widder, Uulke A van der Heide, Danny Schuring, Suresh Senan

**Affiliations:** 1 Department of Radiation Therapy, Catharina Hospital, Eindhoven, The Netherlands; 2 Department of Radiation Oncology, VU University Medical Center, Amsterdam, The Netherlands; 3 Department of Radiation Oncology, University Medical Center Groningen, Groningen, The Netherlands; 4 Department of Radiation Oncology, University Medical Center Utrecht, Utrecht, The Netherlands

## Abstract

**Background:**

A phase III multi-centre randomised trial (ROSEL) has been initiated to establish the role of stereotactic radiotherapy in patients with operable stage IA lung cancer. Due to rapid changes in radiotherapy technology and evolving techniques for image-guided delivery, guidelines had to be developed in order to ensure uniformity in implementation of stereotactic radiotherapy in this multi-centre study.

**Methods/Design:**

A Quality Assurance Working Party was formed by radiation oncologists and clinical physicists from both academic as well as non-academic hospitals that had already implemented stereotactic radiotherapy for lung cancer. A literature survey was conducted and consensus meetings were held in which both the knowledge from the literature and clinical experience were pooled. In addition, a planning study was performed in 26 stage I patients, of which 22 were stage 1A, in order to develop and evaluate the planning guidelines. Plans were optimised according to parameters adopted from RTOG trials using both an algorithm with a simple homogeneity correction (Type A) and a more advanced algorithm (Type B). Dose conformity requirements were then formulated based on these results.

**Conclusion:**

Based on current literature and expert experience, guidelines were formulated for this phase III study of stereotactic radiotherapy versus surgery. These guidelines can serve to facilitate the design of future multi-centre clinical trials of stereotactic radiotherapy in other patient groups and aid a more uniform implementation of this technique outside clinical trials.

## Background

Until recently, conventionally fractionated high-dose radiation therapy was the preferred treatment in patients with stage I NSCLC who were unfit to undergo surgery or declined surgery. This is, however, a poor alternative to surgery in operable patients as the mean reported crude local recurrence rates are as high as 40% (range 6–70%), resulting in a three year overall and cause-specific survival of only 34 and 39%, respectively [1].

Recently, stereotactic radiotherapy has gained much interest in the treatment of medically inoperable patients with stage I lung cancer, as local control rates are dramatically improved with this technique compared to conventional fractionation. In studies where schedules with a biologically effective dose (BED) larger than 100 Gy are used, typical local control rates are approximately 90%. The largest series were reported from Japan [2,3], United States [4] and the Netherlands [5], comprising experience in over 750 patients. Onishi et al. [6] retrospectively described the results of 257 patients treated in 14 Japanese centres using a number of different fractionation schedules and delivery approaches. This Japanese study also included nearly 100 patients who refused surgery, and the 5-year overall survival rate of 70.8% observed after a BED of 100 Gy among those patients is at least equivalent to the outcome after surgery [7-9]. Currently, several phase II trials have started in operable lung cancer patients [10] (RTOG 0618 and JCOG 0403), however, to date no prospective multi-centre randomized studies have been performed to compare stereotactic radiotherapy with surgery in patients with operable lung cancer.

**Figure 1:**
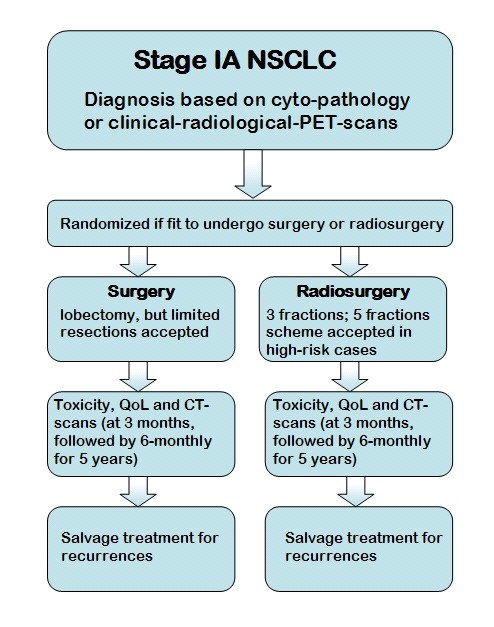
**ROSEL study design**.

A randomized phase III trial of Radiosurgery Or Surgery for operable Early stage (stage 1A) non-small cell Lung cancer (ROSEL, ClinicalTrials.gov ID = NCT00687986) has been opened for accrual in August 2008. The study is initiated by the VU medical centre Amsterdam and the Dutch Lung Cancer Research Group. The primary study objectives are to compare local and regional control, quality of life and treatment costs at 2 and 5 years in patients who are randomized to either surgery or radiosurgery (Figure 1). Treatment costs are a primary end-point, as the costs associated with surgery for stage IA in The Netherlands are far higher than the present costs of stereotactic radiotherapy [11]. These costs are expected to be even more if the costs of post-operative revalidation and loss of economic activity are taken into account. However, patients treated with stereotactic radiotherapy could incur costs for salvage treatment if a higher incidence of local or regional recurrences is detected. Therefore, treatment costs were considered to be a relevant end-point.

Secondary objectives include overall survival, pulmonary function tests, quality adjusted life years and total costs (both direct and indirect). In case of surgery, a lobectomy should be carried out, but limited resections are acceptable. Careful radiological follow-up is performed within the trial in patients treated by SRT, as salvage surgery or mediastinal radiation therapy might still be possible in case of clinical, radiological or histological evidence of local or hilar disease progression.

Accreditation and dosimetry guidelines have been previously developed for trials of stereotactic radiotherapy such as RTOG 0236 and JCOG 0403 [12-14]. However, a reassessment was considered necessary because a new patient group was being treated with stereotactic radiotherapy, namely patients who were fit to undergo both primary and salvage surgery. As a result, normal tissue dose-constraints had to be more stringently defined in order to minimize the risk of increased complications after salvage surgery. Furthermore, IGRT technology from different vendors has been rapidly adopted at various Dutch centres, which had to be taken into account. The resulting guidelines include both minimum requirements that must be met by each participating centre as well as recommendations for possible further improvements. They are presented here in order to facilitate the implementation of future multi-centre studies, to stimulate and improve the implementation of stereotactic techniques in clinical practice and to improve the comparability of results.

## Methods

A ROSEL Quality Assurance Working Party was formed by radiation oncologists and medical physicists from both academic as well as non-academic hospitals that had already implemented stereotactic radiotherapy for lung cancer. Several working party meetings were organised in which both the knowledge from literature and clinical experience were shared and amalgamated. In support of these meetings, a literature search was conducted by searching MEDLINE with different key words and their permutations such as stereotactic radiotherapy, stage I lung cancer, treatment planning, CT scan, patient positioning and tumour mobility. Abstract books of the ASTRO, ASCO, AAPM and ESTRO/ECCO from 2004 to 2008 were reviewed. It was recognized that there was little data available in the literature about the influence of different planning algorithms on the planning of stereotactic radiotherapy. Therefore, an additional planning study was performed in 22 stage IA and 4 stage 1B non-small cell lung cancer patients in order to develop and evaluate the planning guidelines differentiated according to type of dose calculation algorithm used. Patient characteristics and treatment planning details have been reported previously [15].

In brief, a four-dimensional (4D)-CT was reconstructed in ten equally spaced time bins using respiratory phase binning for each patient. From these phases, a maximum intensity projection (MIP) was reconstructed [16]. The datasets were then imported in the Pinnacle^3 ^treatment planning system (Philips Medical Systems, Wisconsin). Using the MIP dataset, an experienced radiation oncologist delineated the internal target volume (ITV). Organs at risk were delineated on an average-density CT reconstruction. The PTV was created by expanding the ITV with a 3 mm margin. The treatment plans consisted of 9 equally spaced coplanar 6 MV beams which were not allowed to enter through the oesophagus, heart, spinal cord or contralateral lung. The plans were inversely optimized using the direct aperture optimization module of the Pinnacle^3 ^treatment planning system with the same objectives as used in the ROSEL trial. Three different plans were created; using an advanced (type A) dose calculation algorithm, a less advanced (type B) algorithm and a plan assuming all tissues within the body to have unit density, in accordance with the RTOG study 0236 and 0618 protocols [17,18].

In order to facilitate the clinical use of these recommendations, we divided the process of implementing high-dose radiotherapy into the following headings: CT scanning and patient positioning, target volume definition, organs at risk definition, Dose calculation algorithms and fractionation, dose prescription, coverage and constraints, treatment planning and treatment execution.

## Patient positioning and CT scanning

The patient should be scanned in the treatment position which should be supine with both arms raised above the head using an arm-rest or other fixation device. Positions which are less comfortable for the patient should be avoided so as to prevent the likelihood of uncontrolled movement during scanning or treatment. Four-dimensional (4D) CT scanning is strongly recommended in order to account for an individualised assessment and incorporation of tumour motion [19-21]. Preferably 10 but no less than 6 breathing phases should be reconstructed in order to determine the tumour movement for treatment planning. Using 10 phases, it was found that generally the full amplitude of motion can be captured [22]. Within the ROSEL trial, acquisition of a slow-CT scan or multiple (at least 3) rapid planning scans covering the entire range of tumour motion is also allowed, as 4D-CT scanners are not widely available yet. However, target volume delineation might be more difficult as the images, and thus also the tumour volume, of slow-CT scans are blurred [23,24]. All centres participating in the ROSEL study will most likely be able to implement 4D-CT scanning in the near future. Generally, intravenous contrast is not necessary for planning CT scans for early stage lung cancer, but contrast-enhanced CT images may still be used for dose calculations. Although the effect of intravenous (IV) contrast on dose calculations for lung patients is not specifically studied, the influence of IV contrast in head and neck intensity modulated radiotherapy plans was proven to be insignificant [25]. The slice spacing between reconstructed CT images should be ≤3 mm over the complete tumour trajectory and ≤5 mm elsewhere. The scan should encompass the entire lung volume in order to calculate meaningful lung dose-volume parameters.

## Target volume definition

The gross tumour volume (GTV) will generally be contoured using CT pulmonary windows; however, soft tissue windows may be used to avoid inclusion of adjacent vessels or chest wall structures within the GTV. The correctness of the GTV delineation should be checked in axial, sagittal and coronal views. The clinical target volume (CTV) is assumed to be identical to the GTV, i.e. with no margin for microscopic disease added, which appears to be justified by the high local control rates observed in patients undergoing careful post-treatment follow-up [26]. This approach has also been accepted in the ASTRO-ACR recommendations on stereotactic radiotherapy [27].

For PTV definition, two main treatment planning and execution techniques can be distinguished; planning and irradiation based on the internal target volume (ITV) concept or the time-averaged mean position of the tumour.

### PTV based on the ITV concept

For 4D CT scans, the ITV can be derived from the union of GTV delineations on all breathing phases or alternatively, from contouring on a maximum intensity projection (MIP) CT-dataset [28,29]. The appropriateness of the MIP-delineation should at least be confirmed by a visual inspection of the projected ITV contours on the CT-datasets of the end-inspiration and end-expiration phase bins using axial, sagittal and coronal views. In addition to the MIP contouring, the GTV should also be contoured in a single phase (preferably the end-expiration phase, because this is the most stable tumour position and the phase with the least breathing artefacts) in all patients in order to determine the GTV size. For checking the ITV contour based on the MIP it is not necessary to delineate the end-inspiration and end-expiration phase bins (visual assessment suffices). Alternatively, the ITV may be constructed by the union of all delineations of the GTV in all breathing phases. If only 3D CT data is available, the ITV should be based on either multiple slow CT-scans covering the whole tumour trajectory or an additional margin of 3–5 mm in all directions around the CTV determined on a single slow CT-scan [30]. The ITV to PTV margin is primarily meant to take into account patient set-up uncertainties. However, small intra-fractional variations in the tumour motion and mean position may be present. Also inter-fractional variations may be present, but these might be corrected for using tumour based image guided position verification and correction [31]. In addition, small delineation uncertainties will exist. Thus, a minimum of 3 mm ITV to PTV margin is required in all dimensions, even if a set-up error of <3 mm can be guaranteed. On the other hand, the ITV to PTV margin should not exceed 5 mm, as this would unnecessarily enlarge treated volumes. In case an institution would need to apply a larger margin, e.g. because of their set-up accuracy, it is advised to first improve its (set-up) technique (see also paragraph about treatment execution).

### PTV based on the mean tumour position

As an alternative to the ITV concept, planning and irradiation based on the time-averaged mean position of the tumour has been developed [32]. In contrast to the ITV to PTV margin discussed previously, the CTV to PTV margin needed here should take the tumour motion into account. However, similar to the reasoning given for the ITV to PTV margin, a minimum margin of 3 mm should be used for the incorporation of the other uncertainties.

## Organs at risk definition

Dose volume criteria for organs at risk (OAR) given in a next paragraph are all constraints to the highest doses received by the OAR. As a consequence, the impact of differences in delineation protocols between institutions is not expected to be high, as these differences are likely to be primarily of influence on the delineations located outside the high dose region. However, in order to support future normal tissue complication probability (NTCP) modelling studies, the OAR delineation guidelines as used in the ROSEL protocol are given below.

When 4D-CT scans are used for treatment planning, the critical OAR should be contoured on the relevant reference reconstruction (i.e. the scan used for dose calculations, see also paragraph about treatment planning). This can generally be performed without taking into account potential mobility of these organs, as current experience is based on this type of delineations. However, extremes of motion of organs such as the oesophagus may influence the choice of beam arrangements in case of 'peripheral' lesions located in the proximity of the mediastinum [33]. Also, patient set-up corrections due to tumour shifts lead to a change in the dose given to the OAR. To avoid excessive doses to OAR, it is recommended to evaluate the impact of such shifts on the OAR dose during treatment planning. This might be accomplished by using Planning organ at Risk Volumes (PRV) [34].

The spinal cord and oesophagus should be contoured starting at least 10 cm above the superior extent of the PTV and continuing on every CT slice to at least 10 cm below the inferior extent of the PTV. For patients with tumours located in the mid- or lower zones of the lungs, the pericardium and/or heart should be contoured as a single structure. The superior aspect (or base) for purposes of contouring will begin at the level of the inferior aspect of the aortic arch (aorto-pulmonary window) and extend inferiorly to the apex of the heart.

The defined ipsilateral brachial plexus originates from the spinal nerves exiting the neural foramen on the involved side from around C5 to T2 [35,36].

For peripheral tumours in the upper lobes, the major trunks of the brachial plexus should be contoured, using the subclavian and axillary vessels as surrogates. This neurovascular complex will be contoured starting proximally at the bifurcation of the brachiocephalic trunk into the jugular/subclavian veins (or carotid/subclavian arteries) and following along the route of the subclavian vein to the axillary vein ending after the neurovascular structures cross the 2nd rib.

The trachea and proximal bronchial tree are contoured as two separate structures using mediastinal windows on CT to correspond to the mucosa, submucosa and cartilage rings and airway channels associated with these structures. For this purpose, the trachea will be divided into two sections: the proximal trachea and the distal 2 cm of trachea. The proximal trachea will be contoured as one structure, and the distal 2 cm of trachea will be included in the structure identified as proximal bronchial tree (main carina, right and left main bronchi, right and left upper lobe bronchi, intermedius bronchus, right middle lobe bronchus, lingular bronchus, right and left lower lobe bronchi).

Delineation of the chest wall has not been regularly performed. Little is known about chest wall morbidity in relation to dose in stereotactic radiotherapy, and therefore delineation is not mandatory within the ROSEL trial [37]. However, it is recommended to delineate the chest wall in case of tumours in close proximity to the chest wall. This will aid the development of NTCP models concerning chest wall toxicity.

## Dose calculation algorithms and fractionation

A number of different dose fractionation schedules have been reported for lung SRT [38,39], but the optimal dose fractionation schedule may vary with tumour stage and location. Although no randomized studies comparing different fractionation schedules have been conducted for stage I tumours, most of the clinical experience is based on schedules with 3 fractions of 20 Gy. In RTOG study 0236, RTOG study 0618 and in the ROSEL study, this fractionation scheme is used. In all studies, eligibility for inclusion was limited to lesions located ≥ 2 cm distal to the hilar structures. Within the ROSEL study, a more conservative fractionation scheme of 5 fractions of 12 Gy is also allowed for patients with a tumour with broad contact to the thoracic wall or adjacent to the heart or mediastinum. Lung function is not considered to affect the scheduling or fractionation. The largest clinical experience published thus far did not exclude any patient on the basis of poor lung function [26], and did not observe excessive lung toxicity when 'risk-adapted' SRT schemes were used This is supported by 2 recent reviews [40,41]. A report by Timmerman [42] which suggested that toxicity rates were high for central tumors treated with SRT has been criticized on the grounds of the toxicity definitions used [43].

However, it is recognized that differences between calculation algorithms in the various treatment planning systems may be as high as 30% in individual cases [15]. These differences are largest for lung tumour treatment plans, and generally increase with decreasing field size, which is especially relevant in stereotactic radiotherapy of stage 1A lung tumours. Thus, depending on the treatment planning algorithm used, one should actually use an alternative nominal fraction dose to deliver the same actual dose to the patient. Unfortunately, extensive data comparing all the calculation algorithms that are likely to be used in the ROSEL study are not available. For the nominal dose fractionation schedules allowed within the ROSEL trial two main type of algorithms are distinguished [15,44].

• Type A models: Models primarily based on electronic path length (EPL) scaling for inhomogeneity corrections. Changes in lateral transport of electrons are not (well) modelled. The algorithms in this group are e.g. Eclipse/ModBatho and Eclipse/ETAR from Varian, OMP/PB and Plato/ETAR from Nucletron, PrecisePLAN from Elekta, I-plan Dose/PB from BrainLAB, and XiO/Convolution from CMS.

• Type B models: Models that in an approximate way consider changes in lateral electron transport. The models in this group are e.g. Pinnacle/CC from Philips Medical Systems, Eclipse/AAA from Varian, OMP/CC from Nucletron, I-Plan-dose with XVMC Monte-Carlo algorithm from BrainLAB and XiO/Superposition from CMS.

As a guideline, the fractionation schedule(s) and dose constraints one wants to implement should be adapted to the dose algorithm used. For example, within the ROSEL trial, it was decided that for type A models, a standard fractionation schedule of 3 fractions of 20 Gy or 3 fractions of 18 Gy and a conservative fractionation schedule of 5 fractions of 12 Gy or 5 fractions of 11 Gy could be allowed. For type B models, the standard fractionation should be 3 fractions of 18 Gy and the conservative fractionation should be 5 fractions of 12 Gy or 5 fractions of 11 Gy. A 3 fractions of 20 Gy schedule is not allowed in combination with type B models in the ROSEL trial, as this might lead to dose levels being approximately 10% higher than the dose levels with which extensive experience has been gained in the VU Medical Centre Amsterdam, using a type A algorithm. These higher dose levels might lead to increased morbidity. The fractionation of 5 times 12 Gy is still allowed with type B models since the errors of type A algorithms in calculating dose to the thoracic wall, heart or mediastinum are expected to be less significant. Although this also would lead to approximately 10% higher dose levels, the biologically effective dose for the PTV will still be well below the BED of the 3 fractions schedule. There are no indications in the literature that this would lead to an unacceptable level of morbidity. It is highly recommended to include dose algorithm specifics in future reports about stereotactic radiotherapy for lung tumours. If a more accurate algorithm becomes available to the authors of such articles, one should also consider the publication of the recalculated data. These data can be used to improve our dose-effect models, which aid the further improvement of stereotactic radiotherapy.

## Dose prescription, coverage and constraints

In line with current multi-institutional trials and multiple single-centre experiences, the dose prescription should be based on 95% of the target volume (PTV) receiving at least the nominal fraction dose (e.g., 20 Gy per fraction = 60 Gy total), and 99% of the target volume (PTV) receiving a minimum of 90% of the fraction dose. The dose maximum within the PTV should preferably not be less than 110% or exceed 140% of the prescribed dose, similar to the criteria formulated in RTOG protocol 0618 [18]. The location of the treatment plan normalization point, which is in fact only influencing the display of the dose distribution, can be left to the institutions preference.

RTOG trial 0236 defined a set of parameters to quantify the conformity of the dose and PTV coverage. The same parameters were used in RTOG trial 0618 and are used here. However, the ROSEL trial requires the use of inhomogeneity corrections, whereas this is not allowed within the RTOG trials. Consequently, the dose conformity requirements in the ROSEL study differ from the RTOG recommendations. Moreover, a distinction in these values is made between type A and B algorithms, because of the significant differences in calculation results between them (Table 1).

**Table 1:** Dose conformity requirements and definition of protocol deviations. R_100% _and R_50% _= ratio of respectively the 100% and 50% Prescription Isodose Volume to the PTV. D_2 cm _= dose maximum at 2 cm from the PTV as percentage of the prescribed dose. V_20 Gy _= Percent of lung receiving 20 Gy or more (both lungs minus GTV).

Type A models (standard algorithms)								
R_100%_		R_50%_		D_2 cm _(%)		V_20 Gy _(%)		PTV (cc)
Deviation		Deviation		Deviation		Deviation		
None	Minor	None	Minor	None	Minor	None	Minor	
				
<1.15	1.15–1.25	<8	8–10	<55	55–60	<4	4–6	0–20
<1.15	1.15–1.25	<7	7–8	<65	65–70	<6	6–8	20–40
<1.10	1.10–1.20	<6	6–6.5	<65	65–75	<8	8–10	>40
								
Type B models (more advanced algorithms)								
R_100%_		R_50%_		D_2 cm _(%)		V_20 Gy _(%)		PTV (cc)

Deviation		Deviation		Deviation		Deviation		
None	Minor	None	Minor	None	Minor	None	Minor	
				
<1.25	1.25–1.40	<12	12–14	<65	65–75	<5	5–8	0–20
<1.15	1.15–1.25	<9	9–11	<70	70–80	<6	6–10	20–40
<1.10	1.10–1.20	<6	6–8	<70	70–80	<10	10–15	>40

**Figure 2:**
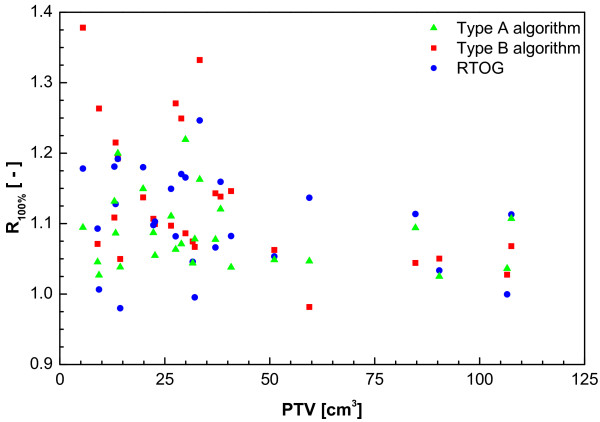
**Ratio of Prescription Isodose Volume to the PTV (R_100%_) from a total of 22 patients with stage IA tumours and 4 patients with stage 1B tumours (with PTVs of 59 cc, 85 cc, 107 cc and 108 cc)**.

**Figure 3:**
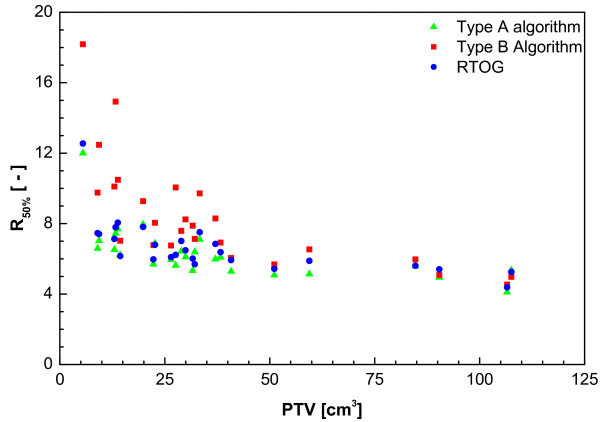
**Ratio of 50% Prescription Isodose Volume to the PTV (R_50%_) from a total of 22 patients with stage IA tumours and 4 patients with stage 1B tumours (with PTVs of 59 cc, 85 cc, 107 cc and 108 cc)**.

**Figure 4:**
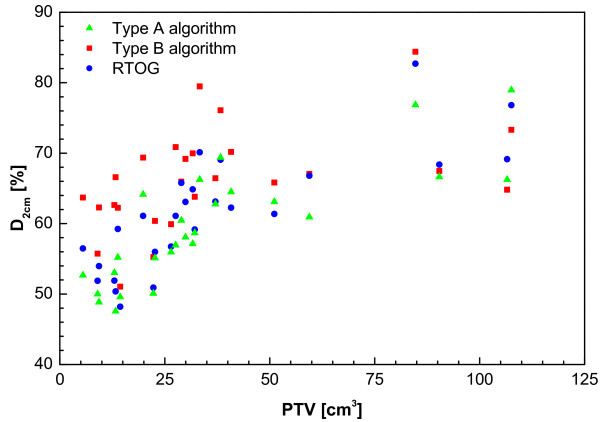
**Maximum dose 2 cm from PTV in any direction (D_2 cm_) as % of prescribed dose from a total of 22 patients with stage I tumours and 4 patients with stage 1B tumours (with PTVs of 59 cc, 85 cc, 107 cc and 108 cc)**.

**Figure 5:**
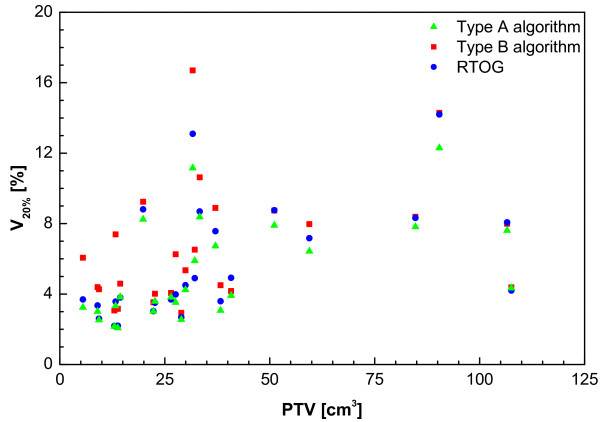
**Percent of lung (both lungs minus GTV) receiving 20 Gy or more (V_20 Gy_) from a total of 22 patients with stage I tumours and 4 patients with stage 1B tumours (with PTVs of 59 cc, 85 cc, 107 cc and 108 cc)**.

**Figure 6:**
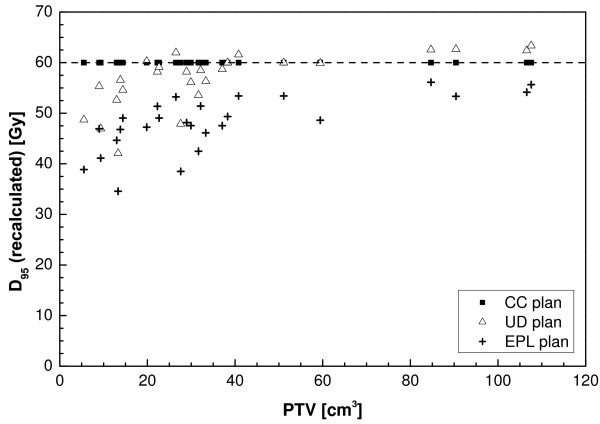
**Dose to 95% of the PTV as a function of the PTV after recalculation using a type B algorithm (Collapsed Cone (CC) algorithm, Pinnacle 8.0 h) from a total of 22 patients with stage IA tumours and 4 patients with stage 1B tumours (with PTVs of 59 cc, 85 cc, 107 cc and 108 cc) (reprinted with permission from ref 20)**. Plans were optimized using a type A algorithm (EPL), a unit density calculation (UD) or a type B algorithm (CC).

From Figure 2 it is clear that using a type B algorithm, it is more difficult to conform the planned dose to the PTV than using a type A algorithm, especially for a small PTV. This is caused by the increased influence of lateral scatter disequilibrium for smaller PTV, which is modelled better using a type B algorithm. Thus, a less strict conformity requirement was formulated. The difference between type B and type A or unit density calculations is even more pronounced for the R50% values (Figure 3). Also for the dose at 2 cm from the PTV (Figure 4) and the percentage of the lung receiving more than 20 Gy (Figure 5), it is clear that a type B algorithm will result in higher values, due to the fact that the change in lateral scattering in lung tissue is taken into account much better. Again, the conformity requirements for type B algorithms were relaxed for these parameters. However, relaxation of these requirements does not result in an actual inferior patient treatment. On the contrary, because these more advanced algorithms provide a better description of the actual dose distribution, the user has a greater opportunity to optimize the dose distribution to the stated requirements. Therefore, the use of these more advanced algorithms is strongly encouraged. Please note that the figures presented here are based on the treatment plans generated without recalculation with a more advanced algorithm, thus representing treatment planning clinical practice within the ROSEL trial, while in the article of Schuring and Hurkmans the results were presented after recalculation, thus quantifying the actual delivered dose differences arising from the use of different algorithms [15]. To emphasize the improvement that can be achieved using a more advanced algorithm over a type A algorithm or a unit density calculation, the dose to the PTV after recalculation is given in Figure 6 (reprinted with permission from Schuring and Hurkmans [15]. The figure clearly shows that The EPL plans (Type A algorithm) consistently overestimate the dose to the PTV, resulting in an average D_95 _of 48 Gy, 20% lower than the prescribed value. The overestimation of the dose increased with decreasing PTV size, although large variations are observed between individual patients. For the unit density calculations the recalculated D_95 _ranged between as much as 63 and 42 Gy for individual patients.

Dose-volume constraints for OAR within the ROSEL protocol are given in Table 2 and differ from the ones used in RTOG 0236 and 0618 (for lung constraints, see previous Table 1). A reassessment was considered necessary because a new patient group will be treated with stereotactic radiotherapy within the ROSEL trial, namely patients who are fit to undergo both primary and salvage surgery. As a result, normal tissue dose-constraints have to be more stringently defined in order to minimize the risk of increased complications after salvage surgery. Additionally, new constraints were formulated to be used for the 5 fraction scheme. Furthermore, the constraints are based on 1 cc volumes (except for the spinal cord), to prevent an excessive dependency on the calculation grid size in the evaluation of these parameters. Skin dose, with the constraint that no point within the skin should receive a dose higher than 24 Gy as dictated in RTOG 0618 is not included in Table 2, as dose calculations within this region are often not very accurate and this dose parameter is often very labour intensive to score. However, this will be evaluated in a dummy run procedure planned before trial participation for each institution.

**Table 2:** Dose constraints for organs at risk and definition of protocol deviations.

Organ	Volume (cc)	Deviation given as cumulative absolute dose (Gy)			
		3 fraction scheme		5 fraction scheme	
		None	Minor	None	Minor
			
Spinal Cord	Any point	18	> 18 to 22	25	> 25 to 28
Oesophagus	1	24	> 24 to 27	27	> 27 to 28.5
Ipsilateral Brachial Plexus	1	24	> 24 to 26	27	> 27 to 29
Heart	1	24	> 24 to 26	27	> 27 to 29
Trachea and main stem bronchus	1	30	> 30 to 32	32	> 32 to 35

## Treatment planning

If treatment planning and irradiation are based on the ITV concept, the PTV incorporates the complete respiratory tumour mobility. Several studies indicate that the use of the ITV concept leads to the use of larger margins than necessary to compensate for tumour motion due to breathing [45-48]. This may in turn lead to the unnecessary exposure of relatively large volumes of organs at risk, especially for patients with very mobile tumours. However, Lagerwaard *et al*. have shown that the incidence of toxicity is low using this concept and a risk-adapted fractionation schedule [26]. Therefore, the use of this concept is accepted within the ROSEL trial. However, one might want to avoid unnecessary exposure of organs at risk due to breathing motion, and four techniques can be distinguished [49]: 1) adaptation of margin recipe [32,50,40], 2) tumour tracking, 3) gating and 4) reduction of breathing motion [51]. These methods are not mutually exclusive, for example, one might use abdominal compression in combination with the mean-position margin recipe. It must be emphasised that introduction of these techniques is not needed for the majority of the patients. In a study performed by Underberg and colleagues, it was shown that only 15% of their patients would have a clinically relevant PTV reduction (defined as 50% or more) using gating compared to the PTV based on the ITV concept [52]. They also showed that the PTV reduction correlated well with the tumour mobility. Thus, the abovementioned techniques should be primarily considered when treating very mobile tumours or for example tumours close to the stomach.

It has been shown that the use of a different margin recipe leads to a similar reduction of the PTV as gating [45,50]. From a patients' perspective, the use of an adapted margin recipe might be preferred, as gating significantly prolongs the treatment time and this, in turn, leads to significantly more intra-fractional changes in tumour position [53]. Also, the use of an abdominal compression plate or active breathing control device might be less comfortable for a patient. This less comfortable position might lead to increased patient movement and no data about this possible effect is available yet. Tumour tracking by means of an external marker does not cause any patient discomfort and might be seen as a patient friendly alternative. However, it is shown that variations in external/internal motion correlation are present, making their use potentially less accurate [54,55]. The use of internal markers is considered more accurate, but is associated with an increased risk of pneumothorax [56]. Furthermore, gating and tracking are also technically challenging techniques. They can only be used on a wide scale if existing technical problems can be solved [57].

Due to the wide penumbra of high energy (≥ 15 MV) beams, it is recommended to only use photon (x-ray) beams with energies of 6–10 MV. Experience has been gained with both coplanar and non-coplanar techniques, with in general a 7–13 beam angles in case static beams are used. Dynamic conformal arcs can be used, although generally thoracic wall doses are larger than with multiple static beams.

For ITV based treatment plans, dose calculations can be performed on the 3D CT scan reconstruction generated without breathing phase binning. (i.e. an average scan or untagged scan reconstruction). This has proven to be a good approximation of 4D dose calculations if combined with a type B algorithm [47,58].

For mid-position based treatment plans, dose calculations should be either performed on the CT reconstruction phase which represents the time-averaged mean position of the tumour or on scan reconstruction generated without breathing phase binning.

## Treatment execution

It is advised to keep the inter-fraction interval at a minimum of 40 hours, in line with the RTOG protocol 0618. The maximum inter-fraction interval should be 4 days. Within the ROSEL trial, the standard fractionation should be given over 5–8 days, while the conservative fractionation should be given over 10–14 days. In general, it is recommended to keep the treatment time as short as possible in order to limit possible patient movement and patient discomfort. Longer sessions have been correlated with significantly more inter-fractional changes in tumour position [53].

Patient positioning should be determined by imaging at the treatment unit itself by means of kV-CT imaging, MV-CT imaging or orthogonal kV imaging. It is strongly recommended that the target position should be compared to the target position in the images used for treatment planning, and appropriate patient set-up corrections should be applied when tumour shifts are detected [31]. As a minimum requirement within the ROSEL protocol, an on-line set-up correction protocol based upon bony anatomy should be applied.

## Discussion

The ROSEL trial Quality Assurance Working Party in this article has tried to present a broad overview of all the technical aspects of stereotactic radiotherapy for early stage lung cancer. Our aim was to develop widely applicable guidelines in view of the number of stereotactic radiotherapy systems used at centres in The Netherlands which will participate in the ROSEL trial. However, we also formulated recommendations assuming the most advanced technical possibilities are at ones disposal. Hopefully, these recommended techniques can be implemented on a large scale in the near future. As stereotactic radiotherapy techniques are in general highly sophisticated, our paper cannot possibly cover all areas in detail. As many aspects of implementation depend on the available equipment, we recommend that centres should familiarize themselves with technical details of the equipment to be used. Appropriate quality assurance systems should also be implemented. A comprehensive overview of quality assurance issues can be found in a special edition about quality assurance of the Int. J. Radiat. Oncol. Biol. Phys. (71S, 2008).

To the best of our knowledge, this is the first trial in stereotactic lung radiotherapy which makes a distinction in dose prescription and dose to OAR criteria based on the calculation algorithm used. As was clearly shown, the dosimetric differences from the use of different algorithms can be large, and it is more difficult to plan a conformal dose distribution using a more advanced algorithm. Without making a distinction based on type of algorithm, this might lead to the incorrect assumption that centres with such algorithms use less conformal techniques. However, it is shown that the actually delivered dose using type A algorithms can deviate as much as 30%, which is highly dependent on the patient specific anatomy and in general the deviation increases with decreasing target volume [15]. Therefore, relationships between treatment outcome and dose generated from stereotactic lung cancer trials which not primarily applied type B calculation algorithms should be interpreted with caution.

## Conclusion

Guidelines and recommendations have been formulated to aid the implementation of stereotactic radiotherapy for early stage lung cancer patients in both individual centres as in future multi-institutional trials. They are formulated such that stereotactic treatment can safely and effectively be implemented in clinical practice in a wide variety of hospitals and treatment results become better comparable.

## Competing interests

The authors declare that they have no competing interests.

## Authors' contributions

CH drafted the manuscript, coordinated and participated in the Quality Assurance Working Party designing the guidelines, and participated in performing the calculations comparing dose calculation algorithms. JC, FL, JW and UH were all members of the Quality Assurance Working Party. DS participated in performing the calculations comparing dose calculation algorithms. SS conceived of the study, and participated in its design and coordination. All authors read and approved the final manuscript.
